# Increase in *Artemisia annua* Plant Biomass Artemisinin Content and Guaiacol Peroxidase Activity Using the Arbuscular Mycorrhizal Fungus *Rhizophagus irregularis*

**DOI:** 10.3389/fpls.2018.00478

**Published:** 2018-04-13

**Authors:** Erzsébet Domokos, László Jakab-Farkas, Béla Darkó, Béla Bíró-Janka, Gyöngyvér Mara, Csilla Albert, Adalbert Balog

**Affiliations:** ^1^Department of Fundamental Pharmaceutical Sciences, Pharmaceutical Botany, Cell Biology and Microbiology, University of Medicine and Pharmacy of Târgu Mureş, Târgu Mureş, Romania; ^2^Department of Electrical Engineering, Sapientia Hungarian University of Transylvania, Cluj-Napoca, Romania; ^3^Department of Horticulture, Sapientia Hungarian University of Transylvania, Cluj-Napoca, Romania; ^4^Department of Bioengineering, Sapientia Hungarian University of Transylvania, Cluj-Napoca, Romania; ^5^Department of Food Science, Sapientia Hungarian University of Transylvania, Cluj-Napoca, Romania

**Keywords:** AMF, plant–fungus interactions, plant biomass, glandular trichome, stress proteins, artemisinin content

## Abstract

The main objective of this study was to investigate *Artemisia annua* plant property variations in terms of plant biomass, glandular trichome numbers, artemisinin production and Guaiacol peroxidase (GPOX) activity when plants are in mutualism with AMF. According to the results, *A. annua* mutualism with AMF significantly increased the most important and pharmaceutically relevant factors of fresh and dry plant biomass. This increase, especially in the biomass of plant herba (leaves), was 30% higher during the vegetation period and remained high (29% higher than for control) when plants were harvested at the end of the vegetation period. Similar differences in dry biomass were also detected. Glandular trichomas numbers increased by 40%, and the artemisinin content by 17% under AMF colonization. No effects due to AMF on chlorophyll variations were detected, while GPOX enzyme concentrations increased significantly under AMF colonization. Altogether the Artemisia plant properties with high pharmaceutically importance (fresh and dry biomass of leaves and artemisinin, number of trichomes and the artemisinin content) were significantly improved by AMF, the application in Artemisia cultivation can be an effective and cheap method. The high GPOX activity under AMF colonization indicate an enhanced oxidative stress alleviation, therefore a higher resistance to water deficiency, mechanisms important under climate conditions with low water supply where Artemisia is usually cultivated.

## Introduction

In developing nations, malaria is one of the most predominant parasitic infections and the 10th overall cause of death. It is estimated that it will remain at that level until 2030 (Mathers and Loncar, 2006; Ikram and Simonsen, 2017; Ikram et al., 2017). According to the World Health Organization (WHO and UNICEF, 2005), more than 380 million cases of malaria occur annually, being responsible for more than 1 million deaths in tropical and subtropical regions (Mathers and Loncar, 2006; Weathers et al., 2011). *Artemisia annua* L. produces plant compounds such as artemisinin, an effective sesquiterpene lactone in malaria treatment, and also in combating other parasitic diseases, certain viral infections and neoplasms (Weathers et al., 2011; Suberu et al., 2013; Stefanache et al., 2016; Ikram and Simonsen, 2017). Based on previous research, the most representative phenolic compounds in *A. annua* are flavones and their glycosides (luteolin, luteolin-7-glucoside, apigenin), flavonols and their glycosides (kaempferol, quercetin, isoquercitrin, rutin, patuletin), coumarins (coumarin, 6,7-dimethoxy-coumarin), and phenolic acids (ferulic acid) (Ivanescu et al., 2010; Stefanache et al., 2016; Ikram et al., 2017). As the compounds in Artemisia plants are in short supply, research on their production and its quantity in plants is of high commercial and medical value – as are low cost methods for drug delivery – moreover its cultivation are practiced under low water supply (Weathers et al., 2011). Although several studies have investigated the artemisinin production of Artemisia plants under different growing systems (Jelodar et al., 2014; Pulice et al., 2016), only a few of them have investigated the effect of arbuscular mycorrhizal funguses (AMFs) effects on these plants (Chaudhary et al., 2008; Awasthi et al., 2011). According to these studies, plants inoculated with *Glomus macrocarpum* and *Glomus fasciculatum* presented an increase in biomass (fresh and dry weight of shoot), concentration of specific nutrients (P, Zn, and Fe) in shoots, and in artemisinin and essential oil content of plants (Chaudhary et al., 2008). Experiment using *Glomus mosseae* and *Bacillus subtilis* together indicated similar results regarding the growth, biomass and the content of artemisinin in *A. annua* plants (Awasthi et al., 2011). Some AMF species, such as *Rhizophagus irregularis* N.C. Schenck and G.S. Sm. (Glomerales: Glomeraceae), are capable to form mutualistic relationship with a wide range of plant species (Kloppholz et al., 2011). Although many of the processes involved in these mutualistic relationships are still unclear, it is known that plant fungus interaction has a positive influence on plants nutrition (absorption of phosphorus and microelements), water management and defense systems (Odebode and Salami, 2004; Hempel et al., 2009; Jiang et al., 2015). Recent studies have also revealed that mutualism is a genetically coordinated recognition process in which synchronized signaling pathways occur between the partners. During mutualism *R. irregularis* produces specific effector proteins that mediate adaptation of the fungus to the conditions present in the root-system (Kloppholz et al., 2011). Another protein secreted by *R. irregularis*, SP7, interacts with the pathogenesis-related transcription factor ERF19 of the plant (Kloppholz et al., 2011). Overexpression of some ERF transcription factors are involved in physiological processes that induce plant resistance to several biotic and abiotic stresses (Lee et al., 2004; Zhang et al., 2009). Little is known about how artemisinin and its metabolites are influenced throughout plant development and in relation to leaf trichomes (Weathers et al., 2006, 2011). During the previous experiments, trichomes densities were analyzed in three types of leaves, in floral buds and flowers, during three developmental stages: vegetative, floral budding, and full flower. According to the results, expression levels in the leaves of early pathway genes, *HMGR*, *PFS*, *DXS*, and *DXR* has not been correlated with either artemisinin or its precursors. Later pathway genes as *ADS* and *CYP*, however, has strong correlation with artemisinin precursor, DHAA, in leaf tissues. A close correlation between artemisinin levels and leaf trichome density was also detected (Weathers et al., 2011). While the *A. annua* cultivation are usually practiced under low water supply conditions, the role of some antioxidant enzyme activation as GPOX under AMF colonization are of high importance. No similar studies on Artemisia were made until now. Very recent studies on carob plants (*Ceratonia siliqua* L.) however demonstrated that AM colonized plants suffered less drought stress because AMF colonization improved plants water status by activating antioxidant enzymes (GPOX) (Essahibi et al., 2018). According to these in the present study, the main goals were to investigate plant property variations regarding plant biomass, glandular trichome numbers, artemisinin production, and antioxidant enzyme activity of *A. annua* plants when these plants are in mutualism with AMF. Our aim was to test the following hypotheses:

(1) Mutualism of *A. annua* plants with AMF *R. irregularis* can increase plant herb biomass.
(2) Plant glandular trichome numbers producing artemisinin can also be increased, thus the quantity of plant compounds (i.e., artemisinin) can also be improved under mutualism with *R. irregularis.*
(3) Antioxidant enzyme activity [Guaiacol peroxidase (GPOX)] may increase under AMF colonization.

## Materials and Methods

### Plant Material and Mycorrhizal Inoculation

**FIGURE 1:**
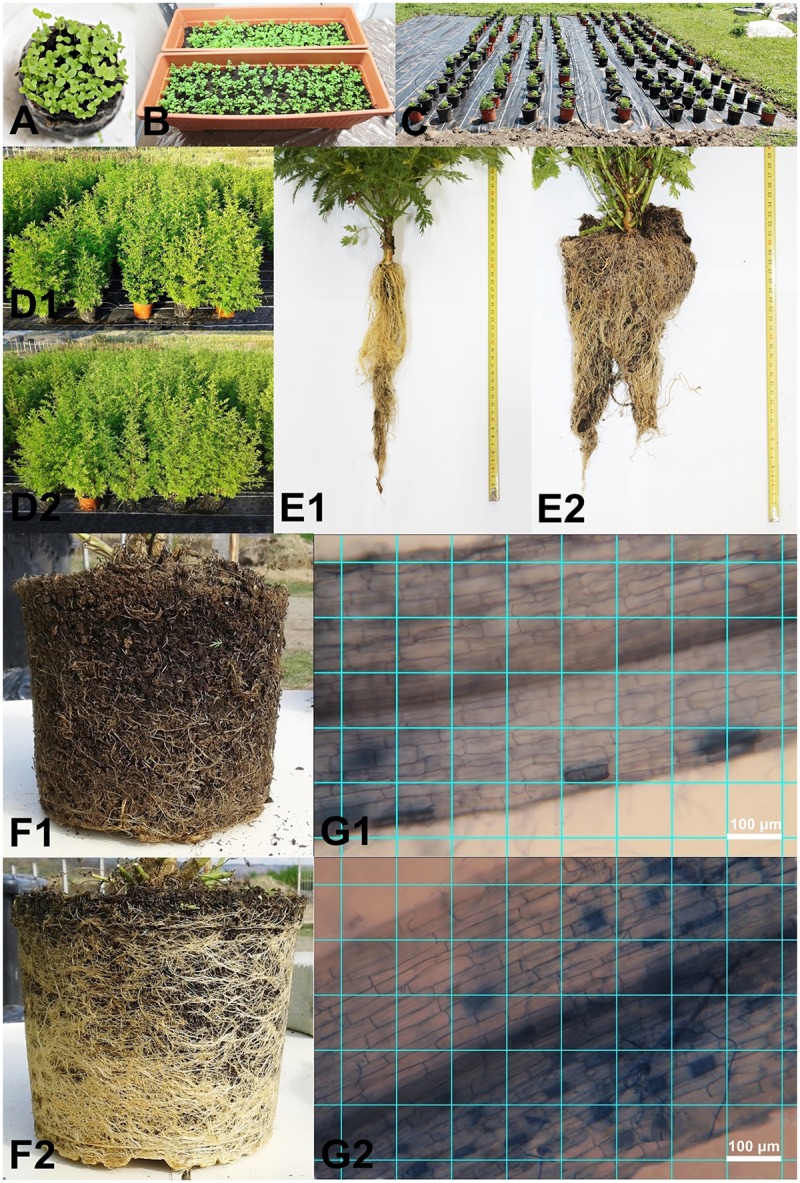
Germinating seeds **(A)**, young plants under lab growing conditions **(B)**, field experiment set-up with blocks **(C)**, control **(D1)** and AMF inoculated plants **(D2)** under vegetation period, root without AMF **(E1)** and root colonized with AMF **(E2)** at the start of the experiment, root without AMF **(F1)** and root colonized with AMF **(F2)** at the middle of the experiment in July and image greed of root colonization without AMF **(G1)** and with AMF **(G2)** (ImageJ Image Processing and Analysis in Java version 1.51j8) (Photos by Erzsébet Domokos).

For this study, seeds of *A. annua* cultivar A-3, from Anamed (Winnenden, Germany) were used. *A. annua* A-3 is a late-flowering hybrid, usually maturing from March to October. It is a high yielding clone that was specially bred to have 20 times the amount of artemisinin found in the wild form. Seeds were germinated in March on peat disks with coconut fiber (Anamed, Germany), under the following laboratory conditions: a temperature of 22°C, air humidity of 40%, 16 h light and 8 h dark photoperiod, 10 ml water/disk/day (**Figure 1A**). The 14 day-old seedlings (in the 2 leaf stage) were transplanted in pots (with 50 cm length, 19.5 height, and 16.3 cm width) containing sterile commercial peat (Blondy Romania SRL) (**Figure 1B**). This growing system was chosen because other research has shown that the effect of *R. irregularis* under open field condition is low or even unobservable, and that the successful establishment of mutualistic interaction with plant roots in field soils depends on the indigenous species present, the species being introduced, and its placement in the soil (Hepper et al., 1988; Engelmoer et al., 2014). When the seedlings reached the 5–6 leaf stage (about 35 day-old seedlings) preparation was started for inoculation with AMF spores following the standard practice. The seedlings were placed separately in 4 l pots with sterile peat and were watered with 400 ml water/day. The inoculation was made with the AMF spores of *R. irregularis* purchased commercially (Italpollina SPA, Italy). Because the product of AMF spores was supplied as a high concentrated powder (1400 spores/g), 0.5 g mycorrhizal inoculum was added to each pot after dissolving in 400 ml water. The pots were not watered in the first 24 h after the AMF treatment. During this period the mutualistic relationship between the plant and AMF could be established. In the subsequent 3 weeks the plants inoculated with AMF were kept separate to those without AMF treatment in a greenhouse for accommodation to daylight.

### Experimental Design

The field experiment was conducted in Corunca, Mureş County (46°31′18.18′′ N and 24°35′53.78′′ E). The plants were moved outside from the greenhouse in early June in pots of 20 l containing sterile peat moss (only dead fibrous materials). This was done in order to test only the effect of AMF on Artemisia plants since under open field conditions the effect of AMF are highly confoundable because of other synergic fungus and bacteria species (Engelmoer et al., 2014). An experimental area of 500 m^2^ had previously been covered with a plastic wrap to avoid contact between the root system of the experimental plants and the soil. A total of 200 plants (100 plants inoculated with AMF and 100 control plants, without AMF treatment) were randomly arranged in a double nested block design. The experiment consisted of eight blocks, each of them comprising 25 plants. Inside each block, the space between the pots in each row was 30 cm, and between the rows 50 cm. The distance between the blocks was 1 m. The entire system was connected to an automatic drip irrigation source (**Figures 1C,D1,D2**). The irrigation was performed with 400 ml of water/pot daily during June and October, and 800 ml/pot daily during July and August. The standard nutrients (N 250, P 50, K 250, Ca 100, Mg 30, Fe 2, Mn 0.3, B 0.3, Cu 0.1, Zn 0.26 ppm) were provided automatically every 2nd week to consistently maintain the treatment regime for both AMF-treated and control plants.

### Root Colonization Analyses

The AMF colonization was evaluated on three randomly selected plants/treatment/block (12 plants inoculated with AMF, and 12 control plants) at early vegetation period 1 week after inoculation (**Figures 1E1,E2**) and in the full vegetation period (8 week old plants) (**Figures 1F1,F2**). A random sub-sample of approximately 150 root fragments (of 1 cm length) per plant was collected. The roots were cleared in 2.5% KOH (90°C) for 45 min, acidified with 1% HCl for 15 min, stained with 0.05% Trypan Blue in acid glycerol (90°C) for 45 min, and then stored in acid glycerol (Koske and Gemma, 1989). From each sub-sample, 10 randomly selected root fragments were mounted in glycerine on slides. The fragments were aligned parallel to the long axis of the slide. Pictures from the root fragments were taken at 200x magnification by a photographic camera (Canon EOS 1100D, Taiwan) mounted on a microscope (Ceti Topic-T, Belgium). One picture, at 200x magnification, covered a field of view with a length of 0.882 mm, and a width of 0.588 mm. For the root fragment, five pictures were obtained. The distance between the pictures was constant for each sub-sample. In the few cases, when the root fragments were too wide to fit into the field of view of the camera, pictures were taken in two width portions (**Figures 1G1,G2**).

### Plant Biomass Measurements

All plants were harvested until the late vegetative stage (in October). The fresh and dry weight of herba (healthy green leaves only) and roots were measured for 72 randomly harvested plants (9 plants/block). This was done in July at the full vegetation stage of all plants. Besides these measurements, the fresh weight of herba and roots for all remaining harvested plants used for the experiment was also determined. Before measurements were made, the plant leaves and roots were washed and dried on blotting paper. After fresh weight measurements, both the shoots and the roots were dried for 3 weeks under shade at an ambient temperature (20°C) and analyses repeated on the dry plants.

### Glandular Trichome Density Assessment

**FIGURE 2:**
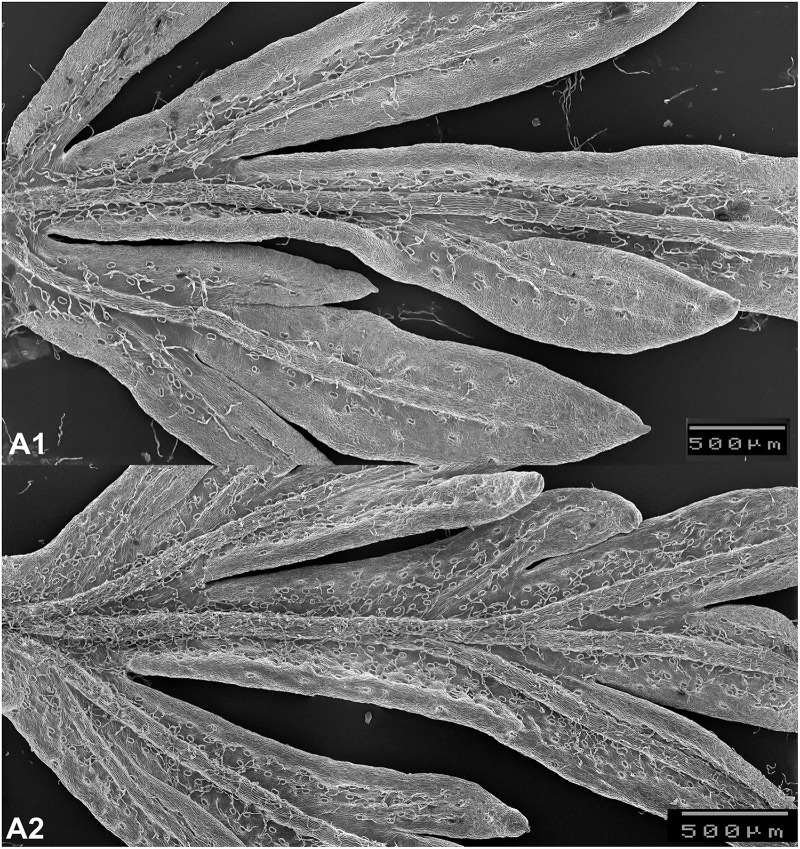
Glandular trichomes on leaves without AMF **(A1)** and with AMF **(A2)** (scanning electron microscope, JEOL JSM-5200) image (Photos by László Jakab-Farkas).

For the determination of glandular trichome density from the upper epidermis of the leaf, a scanning electron microscope (SEM) was used (JEOL JSM-5200, Japan). The SEM was utilized in order to achieve small magnification, high contrast micrographs using secondary electrons at 20 mm working distance, with 1 of 5 kV accelerating voltage as function of sample charging. The fresh leaves of *A. annua* were collected at the full vegetation stage in July and were immediately subjected to analysis with the SEM. For the analysis 10 leaves of 10 randomly selected plants from each block were collected (a total of 80 plants sampled and 400 leaves/treatment analyzed). This represented almost completely the total upper leaves numbers of the plants. The youngest terminal leaf, of about 5 cm in length, on each plant was removed for sampling. The leaf surface was analyzed by starting with a fragment from the leaf mounted on a scanning surface in the form of a square with 0.6 cm sides (**Figure 2**). The number of glandular trichomes were counted with the multi-point tool, while the scanned leaf surface was measured with the polygon selection tool. Then the leaf surface was determined and the number of trichomes reported and averaged in mm^2^.

### Artemisinin Concentration Assessment

A randomly selected 10 plants from each block were used to test the artemisinin content under AMF inoculation and control. Standard artemisinin was purchased from Sigma-Aldrich (Germany). Artemisinin was extracted by refluxing 100 g of dry leaves with hexane at 75°C for 1 h. The hexane was then evaporated under a vacuum and the samples reconstituted in 10 ml acetonitrile then filtered through 0.45 μm syringe filters. The HPLC analyses were performed with Agilent Infinity 1260 on Zorbax Eclipse Plus C18 (100 mm × 3,5 mm), column and detection was conducted at 210 nm wavelength. The acetonitrile: water 65:35% (v/v), was used as a mobile phase with 0.6 ml/min flow rate (Lapkin et al., 2009). The protocol developed by Lapkin et al. (2009) developed and validated an HPLC-RI method and optimized an HPLC-ELSD method. The gradient HPLC-UV method is widely recommended and used for quantification of artemisinin purity and amount in plant material. The calibration curve was constructed by plotting the peak area against the concentration (1.5325, 3.125, 6.25, 12.5, 25 mg/ml) of standard solutions. The determination coefficient (R^2^) was 0.99985.

### Assessment of Chlorophyll Concentrations

The fresh leaves of 20 randomly selected plants (2–3 plants/block) were collected in August. For the spectrophotometric analysis, 0.5 g of fresh plant leaf per plant was used. Buffered 80% aqueous acetone was used for Chlorophyll *a* and *b* extraction according to Porra et al. (1989). The extraction was immediately followed by the absorbance measurement at 730, 664, and 647 nm. Chlorophyll *a* and *b* content was calculated using the following equations (Porra et al., 1989).

$$Chla\;=\;12.25^{*}A^{664}\;-\;2.55^{*}A^{647}$$

$$Chlb\;=\;20.31^{*}A^{647}\;-\;4.91^{*}A^{664}$$

### Guaiacol Peroxidase (GPOX) Enzyme Extraction and Activity Assays

Leaf samples used for enzyme analyses were collected from both AMF*-*treated and control plants on July with full vegetation period, before flowering. Samples were collected randomly from 20 AMF-treated and 20 control plants using the fifth leaves from the top of each plant and counted to have a 500 mg sample/plant. Samples were kept at -20°C until the enzyme extraction and activity assays. For extraction, 200 mg of frozen leaves were homogenized in 1 ml of QB buffer [100 mM KPO_4_ (pH 7.8), 1 mM EDTA, 1% Triton X-100, 10% glycerol, 1 mM DTT (added before use), distilled water], pH 7.8 using a FastPrep Instrument high-speed benchtop homogeniser (MP Biomedicals). The homogenate was centrifuged at 1,000 *g* for 30 min at 4°C, and the supernatant collected. Protein concentration of the enzyme extract was determined by the Bradford method (Bradford, 1976). The activity of GPOX was determined spectrophotometrically at room temperature (20–25°C), at a wavelength of 480 nm in a reaction mixture (1 ml) containing 0.2 mM phosphate buffer (pH 7.5), 20 mM H_2_O_2_ and 20 mM guaiacol 25 μl of crude protein extract. Enzyme activity was determined according to Cavalcanti et al. (2004). One unit of GPOX activity was defined as the amount of enzyme producing 1 μmol of tetraguaiacol per minute. Specific activity was expressed in U/μg protein.

### Data Analyses

The percentage of root colonization by AMF was determined using the program ImageJ Image Processing and Analysis in Java version 1.51j8 (National Institute of Mental Health, Bethesda, MD, United States). On the image a grid of lines was created with an area per point of 0.1 mm^2^. The points were counted in the following categories: with arbuscules, with vesicles, with hyphae only, with fungal material (one or more types of fungal material), with no fungal material, all examined points (**Figures 1G1,G2**). The arbuscular colonization (AC) and the vesicular colonization (VC) were calculated by dividing the number of categories by the total number of examined points. The hyphal colonization (HC) was calculated as the proportion of points with fungal material (**Figures 1G1,G2**). Only proportional differences were calculated between AMF and control plants (no statistical analyses).

Count data obtained from plant biomass, glandular trichomes, and artemisinin content were first tested for normality of errors (Kolmogorov–Smirnov) and for equality of variance (Levene’s test). The plant biomass (fresh and dry herba and root) data, the glandular trichome data and the artemisinin content of leaves were normally distributed. Therefore *t*-tests, following the Student’s *t*-distribution, were used to compare variables (fresh biomass between AMF and control, dry biomass between AMF and control for herba and roots separately). The glandular trichomas exact counts were made using the program ImageJ Image Processing and Analysis in Java version 1.51j8 (National Institute of Mental Health, Bethesda, MD, United States). Then the trichome data between AMF and control plants were compared with the *t*-test. Additionally, *t*-tests (again following the Student’s *t*-distribution) were used to compare the content of artemisinin between AMF and control plants.

Chlorophyll and GPOX data did not meet the assumption of normality (Kolmogorov–Smirnov and Levene’s test) therefore the Wilcoxon signed-rank test was used to compare concentrations between AMF inoculated plants and control plants. Results are presented in table. Linear correlation between the number of trichome and artemisinin concentration were made using Past Program and *r-* and *p*-values calculated.

## Results

### Root Colonization Analyses

Root colonization with AMF of Artemisia plants was successful in all cases. The observations on length and diameter for the young plants (**Figures 1E,E1** control, E2 AMF colonized roots) and for the plants in full vegetation period (**Figures 1F,F1** control, F2 AMF colonized roots) revealed that the root system of AMF-treated plants increased considerably. For the plants inoculated with AMF the AC was 34.75% (*SD*: 10.50), the VC of 2.65% (*SD*: 1.29), and the HC of 50.37% (*SD*: 11.10).

### Plant Biomass Variation Under AMF Colonization

**FIGURE 3:**
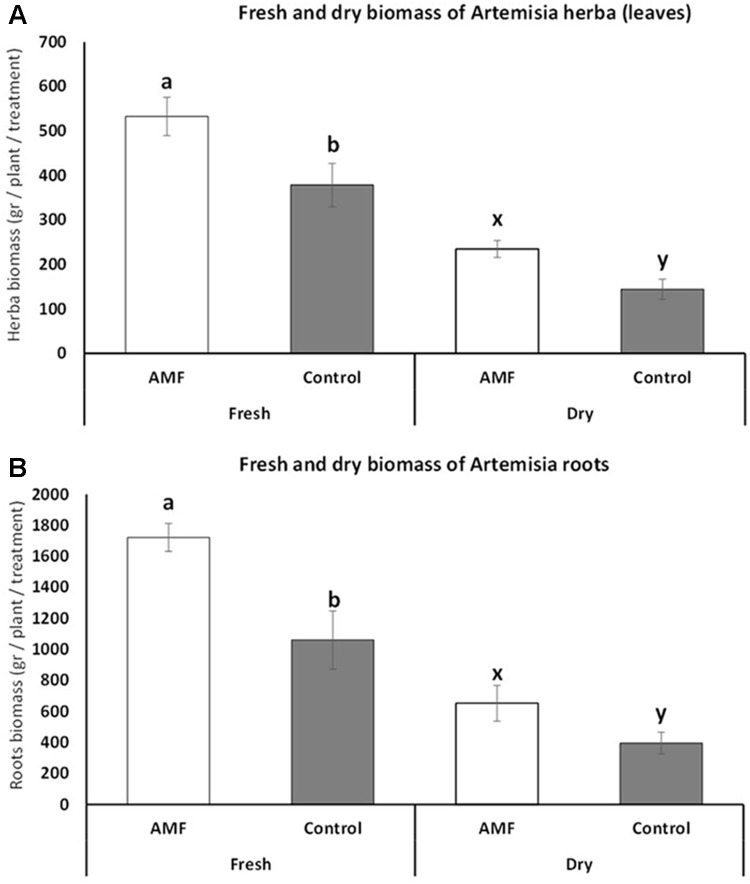
Fresh and dry biomass of *Artemisia annua* herba **(A)** and roots **(B)** under AMF colonization (white bars) and control (without AMF) (gray bars) during the vegetation period (July). *t*-Test following the Student’s *t*-distribution were used to compare variables. Different letters means statistical significant differences at *p* < 0.01 level. Error bars = ± 1 standard error. Data at harvest showed almost the same trend, therefore no additional figures were presented.

The fresh biomass of plant herba at the full vegetation period increased significantly under AMF colonization (*t* = 3.37, *p* = 0.007) (**Figure 3A**). The average fresh biomass increase was 30% higher under AMF colonization, and the same trend was present at dry biomass with an average increase of 40% under AMF and significant differences when compared with the control (*t* = 3.99, *p* < 0.001) (**Figure 3A**). Similarly, the root biomass of AMF plants was also higher for both fresh (average difference of 38%, *t* = 6.38, *p* < 0.001) and dry biomass (average difference of 40%, *t* = 6.39, *p* < 0.001) during the vegetation period (**Figure 3B**). The herba and root fresh biomass at the end of the vegetation period (when the whole 200 plants total herba and root were compared) showed almost the same trend as the data for the vegetation period (average differences at leaf 31%, *t* = 5.72, *p* < 0.001, average differences at root 39%, *t* = 4, *p* < 001). Again the dry biomass shoved the very similar results as during the vegetation period (average differences at leaf 37%, *t* = 5.9, *p* < 0.001, average differences at root 39%, *t* = 4.2, *p* < 0.001).

### Glandular Trichome and Artemisinin Content Variation Under AMF Colonization

**FIGURE 4:**
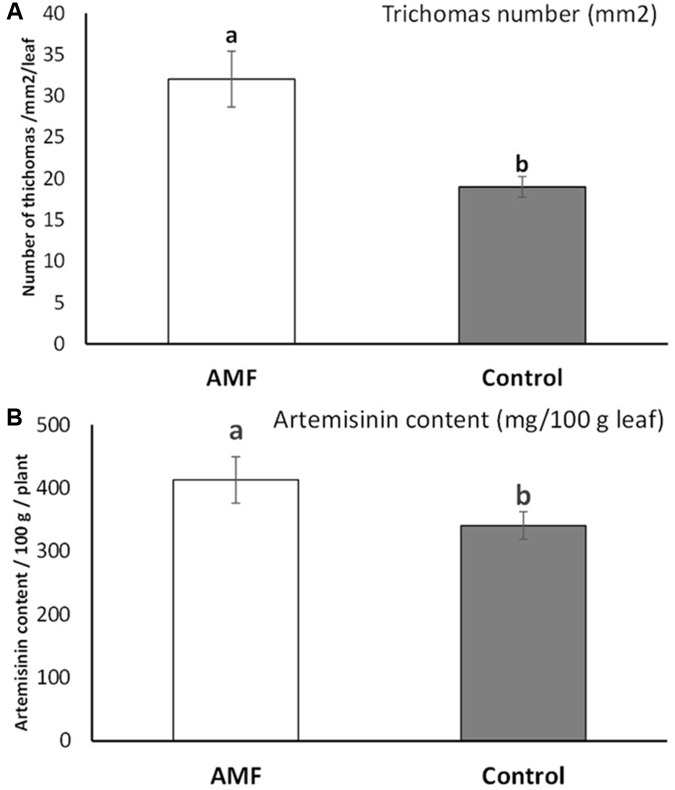
Glandular trichome numbers on *Artemisia annua* leaves (averaged at mm^2^/total leaf) under AMF colonization (white bars) and control (without AMF) (gray bars) **(A)** and the artemisinin content on leaves (mg/100 gm leaves averaged on 10 plants/blocks) of plants under AMF colonization (white bars) and control (without AMF) (gray bars) **(B)**. *t*-Test following the Student’s *t*-distribution were used to compare variables. Different letters means statistical significant differences at *p* < 0.01 level. Error bars = ± 1 standard error.

Substantial increase (an average of 40.7% higher number for AMF plants) and statistically significant differences in glandular trichome number were detected for plants under AMF colonization (*t* = 3.63, *p* = 0.003) (**Figure 4A**). The content of artemisinin also increased significantly (with an average of 17%) under AMF colonization compared to non-treated plants (*t* = 2.13, *p* = 0.04) (**Figure 4B**). Highly significant positive linear correlation (*r* = 0.97, *p* = 0.001) were observed between the number of trichomes and increase in artemisinin content.

### Chlorophyll and Guaiacol Peroxidase (GPOX) Concentration Variations Under AMF Colonization

No differences between AMF and control plants were detected in chlorophyll (both chlorophyll a and chlorophyll b and its percentages). Significantly higher GPOX concentrations were detected on AMF plants comparing with control (**Table 1**).

**Table 1:** Chlorophyll a and b and guaiacol peroxidase (GPOX) concentration of AMF and control *Artemisia annua* plants.

	AMF	Control
Chl.a	*N* = 20	*N* = 20
	Mean: 759.43	Mean: 853.49
	*F* = 1.0898	*p* = 0.28
Chl.b	Mean: 251.34	Mean: 285.72
	*F* = 1.01	*p* = 0.2
Chl a+b	Mean: 1010.8	Mean: 1139.2
	*F* = 1.07	*p* = 0.26
Chl a/b	Mean: 3.0207	Mean: 2.9689
	*F* = 1.14	*p* = 0.54
**Guaiacol peroxidase (GPOX)**		
GPOX U/ml	Mean: 90.12	Mean: 69.29
	*F* = 3.67	***p* = 0.04**
GPOX U/ug	Mean: 4.78	Mean: 3.03
	*F* = 4.11	***p* = 0.04**

*For statistical analyses the Wilcoxon signed-rank test were used. Bold values indicate Statistical significant differences when p < 0.05.*

## Discussion

Based on these results, it is clear that *A. annua* mutualism with AMF significantly increased some important plant properties, such as the fresh and dry biomass of the plants, glandular trichomas numbers, and artemisinin content. Potential factors that may increase plant biomass under AMF colonization need to be considered as *R. irregularis* and other similar AMF species are obligatory biotrophs; in the absence of the host plant they only produce a tiny mycelium that aborts growth after a few days. In the presence of a compatible host plant, the fungus and plant exchange several secreted signals that initiate the symbiotic cellular program (Odebode and Salami, 2004; Bonfante and Genre, 2010; Bonfante and Requena, 2011). Similar studies also revealed that a significant increase in the biomass of *A. annua* was recorded on inoculation with *Rhizophagus fasciculatus* supplemented with P fertilizer. By analyzing the concentrations of mineral nutrients in the leaves of *A. annua* at the end of experiment, revealed that AMF inoculation significantly increased the concentrations of macro- and micronutrients. Also a significant increase in the density of glandular trichome was recorded on AMF inoculated plants and an increase in concentration of artemisinin were detected in AMF plants compared to control. The study also revealed that AMF significantly increase the biomass production and accumulation of artemisinin (Giri, 2017).

The increase in plant biomass by mycorrhizal inoculation is probably due to reduction of leaf nitrogen, thereby increasing membrane stability and concentrations of essential inorganic nutrients such as N, P and K, as also reported by Selvakumar and Thamizhiniyan (2011). Several other plant properties may also be improved under AMF colonization – several control Artemisia plants became yellow at the end of the present experiments, while no discolorations on AMF plants were detected during the whole vegetation period. Decolouration can be explained by viral infection of the control plants but also by low water quantity that might occur at plants with full vegetation stage, cultivated in pots. In other studies, it has also found that AMF colonization highly increased the jasmonate (MeJA) and oligogalacturonic acid, major plant defense signaling compounds conferring additional resistance and yield increase on vegetables such as tomato, potato, and soybean plants (Constabel et al., 1995). Similar research by Selvakumar and Thamizhiniyan (2011) has shown that in saline environment chili plants inoculated with AMF have a higher yield than control plants. Other researches revealed that *R. irregularis*, during its mutualism with vegetable plants, secretes in a very short time (after 20 h) effector proteins that interact with transcription factors (e.g., ERF19) responsible for plant resistance against pathogens. These effector proteins remain active even after 14 days (Kloppholz et al., 2011). The increased resistance can be correlated with upregulation of certain defense-related genes, including PR1, PR2, PR4, Osmotin, and SAR8.2 (Lee et al., 2004; Zhang et al., 2009). The effect of AMF on *Artemisia* plants however has not been previously analyzed.

In our studies a highly significant correlation between trichome numbers and artemisinin content were detected. Similar researches revealed that mycorrhization stimulates the accumulation of artemisinin in plants by enhancing the number of glandular trichome on leafs and also by activating artemisinin synthesis responsible genes (Mandal et al., 2013, 2015). Other factors may also be of high importance in artemisinin production. Mycorrhization induces the methyl erythritol phosphate (MEP) pathway and enhances the level of isopentenyldiphosphate and dimethylallyldiphosphate, two important elements from which artemisinin is built of. It was observed that ibuprofen, an inhibitor of jasmonic acid, reduces the artemisinin concentration in shoots of non-mycorrhizal and mycorrhizal plants. Thus, this enzyme contributes to the mechanism of artemisinin production (Mandal et al., 2015; Ikram and Simonsen, 2017; Ikram et al., 2017). No effect of AMF on chlorophyll has been observed, while significant increase in GPOX activity was detected under AMF colonization by this experiment. The present study thus revealed that the high GPOX activity under AMF colonization may confer Artemisia plants a higher resistance to water deficiency, mechanisms important under climate conditions with low water supply where Artemisia is usually cultivated. According to other studies activation of GPOX were substantially increased in mycorrhizal carob plants under low water supply when experiments on carob plants were made, thereby showing enhanced resistance to oxidative stress induced by drought. Additionally, it has been showed that the effectiveness of AMF colonization in improving carob tolerance to drought was not related to an enhancement of the osmotic adjustment but to an improvement of the GPOX activities (Essahibi et al., 2018). These results are consistent with those previously reported in other plant species as olive, palm, and finger millet (Fouad et al., 2014; Benhiba et al., 2015; Tyagi et al., 2017), but no similar effect on Artemisia has been demonstrated until now.

Altogether the Artemisia plant properties with high pharmaceutically importance (fresh and dry biomass of leaves and artemisinin, number of trichomes, the artemisinin content and GPOX activity) were significantly improved by AMF, the application in Artemisia cultivation can be an effective and cheap method even under low water availability. The effect of AMF on other active ingredients however, has to be tested. The effect of AMF on *A. annua* plants are various and depends on several (probably environmental and biotic) factors that needs to be further considered in order to achieve a practically useful methods for AMF use in Artemisia production.

## Author Contributions

ED, LJ-F, BB-J, and AB performed the field experiments. ED and BD performed the root colonization analyses. ED and LJ-F performed the trichomas analyses. GM and CA performed the chlorophyll GPOX and Artemisinin analyses. ED performed the plant biomass analyses. AB and ED performed the data analyses. AB and ED prepared the figures and tables and wrote the main manuscript text. AB edited and corrected the language of the manuscript. All authors reviewed the manuscript and agreed with submission.

## Conflict of Interest Statement

The authors declare that the research was conducted in the absence of any commercial or financial relationships that could be construed as a potential conflict of interest. The reviewer AM and handling Editor declared their shared affiliation.
